# Efficient reconstruction of biological networks via transitive reduction on general purpose graphics processors

**DOI:** 10.1186/1471-2105-13-281

**Published:** 2012-10-30

**Authors:** Dragan Bošnački, Maximilian R Odenbrett, Anton Wijs, Willem Ligtenberg, Peter Hilbers

**Affiliations:** 1 Department of Biomedical Engineering, Eindhoven University of Technology

## Abstract

**Background:**

Techniques for reconstruction of biological networks which are based on perturbation experiments often predict direct interactions between nodes that do not exist. Transitive reduction removes such relations if they can be explained by an indirect path of influences. The existing algorithms for transitive reduction are sequential and might suffer from too long run times for large networks. They also exhibit the anomaly that some existing direct interactions are also removed.

**Results:**

We develop efficient scalable parallel algorithms for transitive reduction on general purpose graphics processing units for both standard (unweighted) and weighted graphs. Edge weights are regarded as uncertainties of interactions. A direct interaction is removed only if there exists an indirect interaction path between the same nodes which is strictly more certain than the direct one. This is a refinement of the removal condition for the unweighted graphs and avoids to a great extent the erroneous elimination of direct edges.

**Conclusions:**

Parallel implementations of these algorithms can achieve speed-ups of two orders of magnitude compared to their sequential counterparts. Our experiments show that: i) taking into account the edge weights improves the reconstruction quality compared to the unweighted case; ii) it is advantageous not to distinguish between positive and negative interactions since this lowers the complexity of the algorithms from NP-complete to polynomial without loss of quality.

## Background

Techniques for the reconstruction of biological networks, such as genetic, metabolic or signaling networks, are used for getting insight into the inner mechanisms of the cell. They are usually based on perturbation experiments, e.g., gene knockouts or knockdowns, in which one or more network nodes (e.g. genes) are systematically perturbed and the effects on the other nodes are observed. More concretely, in the context of genetic regulatory networks with knockout experiments, the nodes of the networks are genes. Each gene is knocked out at least once and the expressions of the other genes are measured for each knockout experiment. The expression change with regard to the unperturbed wild type defines the influence of the knocked out gene on the other genes. Based on that, connections between the genes can be established. Using the difference in the expression between the perturbed and the wild type, weights and signs can be associated with the connections to quantify the influence and indicate over- and under-expression, respectively.

An important problem in this kind of network reconstruction is that direct connections between genes might be established that are spurious, i.e., do not exist in reality. We illustrate this with the following example. Suppose that the transcription factor *A*, produced by gene *a*, is needed to activate gene *b*. The activation of *b* results in the production of transcription factor *B* which is encoded by *b*. Let *B* on its turn activate gene *c*, which is manifested by the production of the corresponding protein *C*. Also, assume that genes *b* and *c* cannot be activated by any other gene/transcription factor. Now, if we disable gene *a*, for instance by deleting it from the genome, the production of transcription factor *A* is prevented and as a result *B* and *C* will not be produced. Thus, our measurement of the expression of genes *a*, *b*, and *c* will show that *a* directly influences both *b* and *c*. In the reconstructed networks we prefer to directly relate two nodes only if there is a *direct* influence between them. So, a direct connection between *a* and *c*, resulting from the *transitive* influence via *b*, would be obsolete, and the process of removing such indirect influences is called *transitive reduction* (TR) [1].

There are several ways to remove spurious direct relations depending on the representation of the biological networks. For instance, in [2] this is done by comparing the measured influence strength between the direct and indirect interactions, i.e., an interaction chains that do not contain the direct interaction. The direct interaction is removed if it is weaker than the last edge of the indirect one. Similarly, in the genetic networks inference tool Aracne the method of *data processing inequality* is used to remove indirect interactions [3]. This method works for undirected networks. For each triple of genes their pairwise interactions are checked. The one with the lowest score is assumed to be a result of an indirect interaction of the other two. Other approaches to remove obsolete interactions can be found in [4].

Using TR for filtering out spurious connections was proposed by Wagner [5]. The rule is to remove the direct interaction for each gene pair *g* and *g*^*′*^, if there is an alternative chain of interactions between *g* and *g*^*′*^. Going back to our example above, TR would mean that the undesired edge between *a* and *c*, caused by the indirect interaction, is removed from the network.

The first algorithms for TR date from the seventies [1] and there are several other relevant publications on this subject [6,7]. In all previous works on TR the networks were represented as directed graphs without weights on the edges. However, in the network inference algorithms interaction strengths between genes usually play an important role. Motivated by this fact, the concept of TR of weighted graphs was introduced [4,8], where the weights correspond to interaction strengths. Both these papers take interaction signs (promoting or inhibiting) into account.

In [4] several types of TR are described depending on how individual edge weights are extended to paths to quantify the indirect interactions. One of the variants of TR coincides with the definition that we employ in this paper. However, the interaction strength along paths, which is actually used in [4], is defined quite differently from ours. Also, they do not use thresholds in their definitions.

With regard to the definition of transitive closure for weighted graphs and the general theoretical background, the closest to our work is [9]. Their work is also motivated by a biological application, in particular, the analysis of protein-protein interaction networks. The authors define the notion of transitive closure of weighted graphs, but stop short of introducing TR. Originally, we were inspired by their ideas about the transitive measure of interaction along a path in the network.

Unlike the previous work in [4,8], we adopt an approach that disregards the interaction signs. A benefit of this decision is that the TR algorithms are of polynomial complexity and amenable to parallelisation. In contrast, the algorithm in [8] is in the worst case NP-complete, which means that its runtime grows exponentially with the size of the networks. Tests with the Dream challenges [10] show that the omission of signs does not incur any degradation in overall performance compared to the signed weighted TR of [8].

We present parallel versions of the TR algorithms for both unweighted and weighted directed graphs. These algorithms are developed for general purpose graphics processing units (GPUs). GPUs have been extensively used for various applications, including bioinformatics and systems biology. Since GPUs are standard in modern computers, parallel algorithms become an attractive possibility to speed up computations.

The crucial idea of TR on GPUs is to formulate the algorithm in terms of matrix operations. Since GPUs are very efficient in implementing the latter, this results in a remarkable speed-up so that networks consisting of tens of thousands of nodes can be handled within seconds on a standard desktop computer.

Parallel algorithms for computing transitive closure of a graph, which is closely related to TR, have been developed before e.g., [11,12]. However, the only work that we could find that deals explicitly with parallel algorithms for TR is [13]. Their algorithms are restricted to unweighted graphs and claim efficiency for the special case of sparse graphs. To the best of our knowledge, there does not exist a previous implementation of a TR algorithm on GPUs and thus, our work is novel in that sense, too.

### Approach for unweighted graphs

We adopt a graph-theoretic framework to formally represent biological networks. A *directed graphG*=(*V*,*E*) is a pair of sets of *nodesV* and *edgesE*⊆*V*×*V*, i.e., an edge *e*∈*E* is an ordered pair of nodes (*v*,*v*^*′*^)∈*V*×*V*. Without loss of generality, we identify *V* with an arbitrary but fixed order represented by the set of numbers $\underset{–}{n}=\{1,2,\ldots ,n\}$, where *n* is the number of nodes in *V*. For example, in genetic networks, we associate the nodes with genes and the edges with interactions between genes. More formally, a gene *i* is connected to a gene *j* via an edge *e*=(*i*,*j*) if and only if *i* influences *j*. The graph can be equivalently represented by an *adjacency matrixA*. For standard graphs which do not have weights associated with the edges, the elements *A*_*i*,*j*_ of the matrix have value 1, if there is an edge from nodes *i* to node *j* or 0, otherwise.

A *path* from node *i* to node *j* is a sequence *P*_*ij*_=(*k*_0_,*k*_1_,…,*k*_*m*_), where *k*_0_=*i*, *k*_*m*_=*j* and (*k*_*l*−1_,*k*_*l*_)∈*E*, for 0<*l*≤*m*. Nodes *k*_*l*_, where 1≤*l*≤*m*−1, are called *intermediate* nodes. A *cycle* is a path *P*_*ij*_ whose first and last node coincide, i.e., *i*=*j*. A cycle that consists of one edge is called a *self-loop*. A graph is *acyclic* if it does not contain any cycles. We denote the set of edges of *P*_*ij*_with *Edges*(*P*_*ij*_). Let *Paths*(*G*) and *Paths*(*i*,*j*) denote the sets of all paths in the graph *G* and all paths from *i* to *j*, respectively.

#### Definition 1 (**Unweighted Transitive Closure and Reduction**)

The *transitive closure* of a graph *G* is the graph *G*^*T*^=(*VE*^*T*^) with (*ij*)∈*E*^*T*^if and only if there exists a path from *i* to *j* in *G*. The *transitive reduction of an acyclic graphG* is the unique [1] smallest graph *G*^*t*^=(*VE*^*t*^), i.e., with the least number of edges, such that (*G*^*t*^)^*T*^=*G*^*T*^.

**Figure 1:**
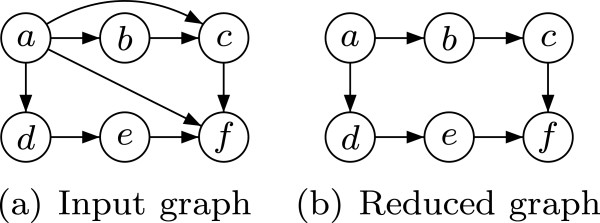
**Transitive reduction of an acyclic graph.** The edge (*a*,*c*) is removed because of the indirect path (*a*,*b*,*c*). Similarly, (*a*,*f*) is removed because of the existence of several indirect paths between nodes *a* and *f*.

Intuitively, this means that the transitive closure is preserved by the reduction, i.e., no information about reachability is lost. For an acyclic graph *G* it can be shown [1] that the TR *G*^*t*^ can be obtained by removing each *redundant* edge (*ij*)∈*E*from the original graph *G* for which there is an indirect path, i.e., not including edge (*ij*), between *i* and *j* in *G*. An example of TR for acyclic graphs is given in Figure 1. 

**Figure 2:**
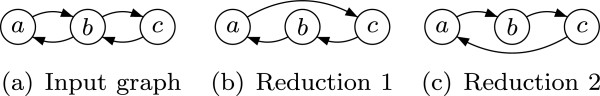
**Transitive reduction of a cyclic graph.** The graphs in **(b)** and **(c)** are both transitive reductions of the graph in **(a)**, since all three graphs have the same transitive closure. One can see that edges that do not exist in the original graph may occur in its transitive reductions, like (*a*,*c*) in (b) and (*c*,*a*) in (c).

The definition of TR can be extended in a natural way to graphs with cycles. However, the reduced graphs are not unique and in general cannot be generated by deleting edges from the graph (see Figure 2). To solve this, Aho, Garey and Ullman [1] shrink the strongly connected components of the graph to single nodes and apply TR on the resulting acyclic graph. After the reduction, we expand the components as they were in the original graph, as is done in [14]. 

### Extension to weighted graphs

We aim at modelling experiments that use node perturbations, e.g., gene knockouts, for the reconstruction of interactions between nodes. We already saw in the example above that spurious direct interactions are added if there exist an indirect path between two genes. Hence, the outcome of the experiments actually produces a transitive closure of the real (original) network. By applying TR as a kind of inverse operation of the transitive closure, we try to cancel these side effects by removing direct interactions between two nodes, if there is an alternative indirect path between them. Finding the TR amounts to reconstruction of the network as by removing those direct interactions we usually obtain a good approximation of the real network.

However, sometimes both direct and indirect interactions can exist at the same time. Examples of this are the feed-forward loops that occur in the genetic networks of many organisms [15]. Unfortunately, in such cases, the TR as defined above will still remove the direct interaction. To avoid this anomaly, we use the notion of TR on *weighted graphs* where the weights represent *interaction uncertainties*. Knowing the interaction uncertainty allows to refine the edge removal criterion: an edge (*ij*) is removed from the original graph only if it is less certain than some indirect interaction between *i* and *j*. In other words, if an edge is at least as certain as all indirect interactions, then it is kept in the graph.

The interaction uncertainties are represented as *weights* of the edges. Formally, we associate to a directed graph *G*=(*V*,*E*) a function w:E→R, which maps each edge to a real number, to obtain a *weighted directed graphG*=(*V*,*E*,*w*). The *adjacency matrixA* becomes a matrix of weights where the special value ⊤>_max*e*∈*E*_{*w*(*e*)} denotes the absence of an edge between two nodes. There are several plausible ways to choose the weight function *w*. In this paper we assume that the weights are *p*-values or similar measures, i.e., of the interval [0,1], which are obtained from the post-processing of the perturbation experiments. Thus, the smaller the weight *w*, the less uncertain is the interaction between two genes.

#### Definition 2 (**(Minimal) Transitive Interaction Uncertainty**)

*Transitive interaction uncertainty* along a path is defined as a function W:Paths(G)→R that maps each path to a real number. We apply the principle of the *weakest link* and define *W*(*P*)=_max*e*∈*Edges*(*P*)_{*w*(*e*)} for a path *P*. Then, for a given edge (*i*,*j*)∈*E* we define the *minimal transitive interaction uncertainty*h:E→R as the strongest weakest link, i.e., the minimal transitive path uncertainty over all paths between nodes *i* and *j*:

$$h(i,j)=\underset{P\in \text{Paths}(i,j)}{\min}\{W(P)\}=\underset{P\in \text{Paths(i,j)}}{\min}\left\{\underset{e\in \mathrm{Edges}(P)}{\max}\{w(e)\}\right\}.$$

Note that if edge (*i*,*j*)∈*E*, then (*i*,*j*)∈Paths(*i*,*j*). This implies directly that *h*(*i*,*j*)≤*w*(*i*,*j*). Recall that the criterion for preserving an edge in the reduced graph is that its uncertainty is not greater than the minimal uncertainty of the indirect paths, i.e., *w*(*i*,*j*)≤*h*(*i*,*j*).

By putting the last two inequalities together, we can refine the edge preservation criterion to *w*(*i*,*j*)=*h*(*i*,*j*) and obtain the following definition:

#### Definition 3 (Weighted Transitive Reduction)

The *transitive reduction of a weighted graph* (TR) *G*=(*V*,*E*,*w*) is the graph *G*^*t*^=(*V*,*E*^*t*^,*w*^*t*^) with *E*^*t*^={*e*∈*E*∣*w*(*e*)=*h*(*e*)} and *w*^*t*^(*e*)=*w*(*e*) for all *e*∈*E*^*t*^.

**Figure 3:**
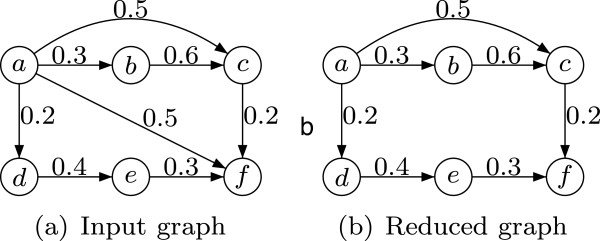
**Transitive reduction of a weighted graph.** Edge (*a*,*f*) with weight 0*.*5 is removed because of the indirect path (*a*,*d*,*e*,*f*) with a smaller transitive interaction uncertainty of 0*.*4. In contrast to Figure 1, the edge (*a*,*c*) is not removed as its weight (0*.*5) is smaller than the transitive interaction uncertainty of the path (*a*,*b*,*c*) (0*.*6).

Informally, edge (*i*,*j*) is kept in *E*^*t*^ if and only if its weight/uncertainty equals the minimal transitive uncertainty of all paths between *i* and *j*. The edge weights remain the same in the reduced graph. It is worth noting that the above definition of TR for weighted graphs supports the presence of cycles. An example of TR for weighted graphs is given in Figure 3.

Of course, there are other possible options to define the path weights based on the edge weights. For instance, summing up the edge weights to obtain the weight of the path is in some cases even a more natural choice than the max-min (weakest link) approach that we use. However, in the case when p-values (or similar metrics in the interval [0,1], e.g., correlation) are used, this is not the best option. Summing up the p-values of all edges in the path can produce a result which is greater than 1, i.e., something which is not a probability. Since a trivial path consisting of only one edge is also a path, it is preferable to have a weight for non-trivial paths that is of the same nature as the edge weight.

In general, the refined notion of TR with weights does not entirely resolve the anomaly of removing a direct interaction which exists in the network. One way to further improve the filtering of the edges is to use thresholds. We introduce a *lower thresholdt*_low_ determining that any edge *e* with *w*(*e*)≤*t*_low_ is unconditionally kept in *E*^*t*^, i.e., regardless of the existence of more certain indirect interactions. In this way we ensure that interactions which are measured with high certainty are not removed from the network. Similarly, we use an *upper thresholdt*_up_ such that any edge *e* with *w*(*e*)≥*t*_up_ is unconditionally removed from the network. Hence, very uncertain connections are always removed from the original graph *G*. This filtering with threshold *t*_up_is actually independent of the TR concept and it can be done as a pre- or post-processing step.

This can be shown by reasoning towards a contradiction. Assume that thresholding (TH) and TR are dependent, in other words, that the final result depends on the order in which TH and TR are performed. Say we have a graph *G* for which this holds. Then, there must be at least one edge *e* from a node *v* to a node *v*^*′*^ which is not present in either TH(*G*^*t*^) or TH(*G*)^*t*^, but it is in the other. Let it not be present in TH(*G*^*t*^) (the case when it is not in TH(*G*)^*t*^is similar). Then, either it was removed by TR (case 1), or by TH (case 2). Case 1: If *e* was removed by TR, there must exist a path *P* in *G* between *v* and *v*^*′*^ with *W*(*P*)<*w*(*e*). The assumption was that *e* is still present in TH(*G*)^*t*^. Therefore, we must have *w*(*e*)<*t*_up_. But then, also *W*(*P*)<*t*_up_, in other words, *P* must exist in TH(*G*). The existence of *P* in TH(*G*) means that *e* must be removed from TH(*G*) when applying TR, leading to a contradiction. Case 2: If *e* was removed by TH, then *w*(*e*)>=*t*_up_. But then, it would also be removed when applying TH on *G*, leading to a contradiction.

Using a threshold *t*_low_splits the edge weights into two sets. Within the sets the difference between values does not play any role. For instance, assuming *t*_low_ = 0.5, the difference between the edge weights 0.8 and 0.7 is the same as between 0.16 and 0.06. An analogous remark holds also for *t*_up_.

**Figure 4:**
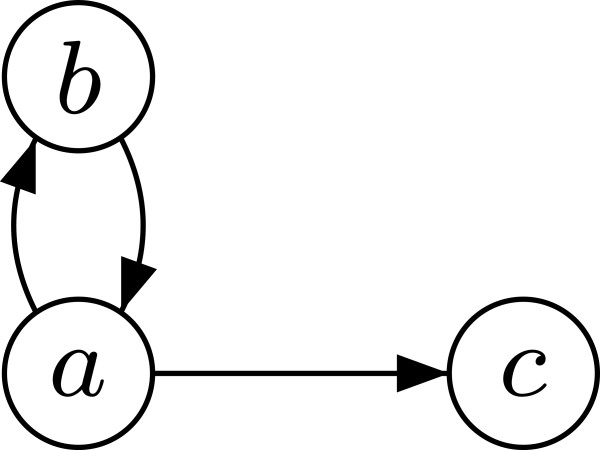
Transitive reduction of cyclic unweighted graphs needs cycle detection.

For many graph problems, the unweighted problem is often a special case of a more general weighted problem. For example, an algorithm to determine shortest paths in a weighted graph can be used to find shortest paths in unweighted graphs by assigning the same positive weight to every edge. For our problem of (weighted) TR, a similar analogy does not hold, i.e., we cannot use the algorithm for weighted cyclic graphs to calculate the TR of an unweighted cyclic graph. This fact results from the different natures of our definitions: For the weighted case, we choose the greatest weight on a path without considering its length. For the unweighted case, however, we will be adding up the edges of paths to obtain their length (cf. next section). The first approach is not affected by cycles – the transitive interaction uncertainty of a path with cycles is always greater or equal to the one for the same path with all cycles removed, thus, cycles are ignored “automatically” when searching a path with the minimal transitive interaction uncertainty. In contrast, for the second approach, cycles must be actively detected to ensure that only paths in which no node occurs more than once are considered (the longest simple path problem is NP-hard). Figure 4 illustrates this with the path (*abac*) which has a length greater than one but still there is no indirect alternative path from *a* to *c*. For a similar reason, our algorithm for cyclic weighted graphs cannot be adapted to cyclic signed weighted graphs. The problems with cycles in signed graphs are discussed in [8]. 

## Implementation

After briefly discussing the emergence of many-core processors and the resulting need for parallelisation of programs, this section presents our parallelised algorithms for transitive reduction: first, for unweighted acyclic graphs and afterwards the extension to weighted cyclic graphs. Finally, we discuss the problems with cycles in unweighted graphs.

### NvidiaCuda GPUs

Although *Moore’s Law*[16], stating that the number of transistors per chip doubles roughly every 18 months, still holds, the clock frequency of the processors does not increase exponentially anymore. Thus, to keep improving the processor performance, the manufacturers turned to processors with multiple *cores*. In particular, General Purpose Graphics Processing Units (GPUs) are an example of massively parallel many-core systems available in desktop computers for an affordable price. However, to benefit from these many cores, programs have to be redesigned for the new architectures.

The *Compute Unified Device Architecture* (Cuda) [17] is Nvidia’s C-based approach to program GPUs. Sequential parts, including input and output operations, are executed “as usual” on the *host* CPU while parallel parts are executed on the GPU *device* by calling a special kind of functions, called *kernels*. The body of a kernel is executed by a configurable number of threads, each having its own ID. The ID determines the data, e.g., part of a matrix in memory, which is processed by the thread. As the GPU device has no access to the host’s main memory, the required data needs to be copied explicitly between them.

### Algorithm for unweighted acyclic graphs

For obtaining the TR of an unweighted acyclic graph *G*, typically its transitive closure *G*^*T*^ would be calculated first. Then, in a second step, all edges *e*=(*ij*)∈*E* would be removed if there exists a node *k*, *k*≠*i*and *k*≠*j*, such that (*ik*)∈*E*^*T*^ and (*kj*)∈*E*^*T*^, i.e., there exists an alternative path (*i*,…,*k*,…*j*) from *i* to *j* in *G* and, since the graph is acyclic, not using *e*. Consequently, *e* is redundant because of the alternative path. The second step is necessary since the transitive closure adds new shortcuts but does not identify shortcuts already existing in *G*. Instead of just calculating the transitive closure, we determine the length of the longest path connecting every pair of nodes (*ij*), *i*≠*j*. This can be done efficiently using a variant of the Floyd-Warshall algorithm [18-20] since the graph is acyclic. Afterwards, we delete all direct edges (*ij*)∈*E*for which a path from *i* to *j* of length at least two exists. Obviously, for this condition the considered lengths can be bounded to two as it does not matter whether the alternative path is exactly of length two or longer.

Algorithm 1 gives a pseudo-code description of our approach. First, the integer-valued adjacency matrix *A* is loaded on the host and copied to the GPU; this copy is called *B* (lines 1–2). Afterwards, in the nested for-loops in lines 3–6, the length of the longest path for all pairs of nodes is calculated by a parallel version of the Floyd-Warshall algorithm. Whenever there is a path of length at least one from *i* to *k*, i.e., *B*_*i*,*k*_≥1, and one from *k* to *j*, i.e., *B*_*k*,*j*_≥1, this gives a path from *i* to *j* of length at least two. Since we are interested only in the existence of an indirect path and not in its actual length, we limit the length of the longest paths to two. Therefore, *B*_*i*,*j*_ is set to two, denoting that there exists an indirect path between *i* and *j*. The last parallel for-loop deletes all edges (*i*,*j*)∈*V*×*V* for which such a detour exists, i.e., *B*_*i*,*j*_=2, by setting *B*_*i*,*j*_:=0 (lines 7–9). Finally, the transitively reduced matrix *B* is copied from the GPU to *A* on the host which stores it to a file.

#### Algorithm 1

**Pseudo-code description of parallelised transitive reduction of unweighted acyclic graphs**Requires: Adjacency matrix *A*

1: read *A* from input file

2: copy *A* from host to *B* on GPU

3: **for***k*=1,…,*n***do sequentially**

4: 

**for**$(i,j)\in \underset{–}{n}\times \underset{–}{n}$**do in parallel**

5: 

**if***B*_*i*,*k*_≥1 and *B*_*k*,*j*_≥1**then**

6: 

*B*_*i*,*j*_:=2

7: **for**$(i;j)\in \underset{–}{n}\times \underset{–}{n}$**do in parallel**

8: 

**if***B*_*i*,*j*_=2**then**

9: 

*B*_*i*,*j*_:=0

10: copy *B* from GPU to *A* on host

11: write *A* to output file

In the Cuda implementation, the parallel do-loop over $(i,j)\in \underset{–}{n}\times \underset{–}{n}$ is realized by starting *n*^2^threads of a kernel containing the loop’s body. Thus, according to its thread ID, each thread executes the loop’s body for one particular $(i,j)\in \underset{–}{n}\times \underset{–}{n}$. In contrast, the sequential do-loop is performed “as usual” by the main program on the CPU.

#### Complexity

The algorithm iterates *n* + 1 times over the *n*^2^elements of the matrix: n times in lines 3–6 and finally once in lines 7–9. However, different steps of the same iteration can run in parallel on different processors. Thus, the overall time complexity depending on the number of processors *p* is: $O\left(\left(n+1\right)*\left\lceil \frac{n^{2}}{p}\right\rceil \right)$. The PRAM model assumes an arbitrary large number of available processors – in particular we can assume *p*≥*n*^2^. In this model, the time complexity becomes *O*(*n*) linear in the number of nodes. For the sequential case, i.e., *p*=1, we have time complexity *O*(*n*^3^) as for the Floyd-Warshall algorithm. Similarly, the space (memory) complexity is *O*(*n*^2^).

#### Correctness

We claim that the output matrix is the TR of the input and prove this for the sequential version, i.e., with sequential execution of the parallel loops, first, before considering the specifics of parallelisation. Thereby we can rely on two things: The correctness of the Floyd-Warshall algorithm (cf. [18]) and the fact that the TR of an unweighted acyclic directed graph can be constructed by successively removing redundant edges from the graph in any arbitrary order ([1], p. 133). It remains to show that our algorithm deletes exactly these redundant edges. An edge (*ij*) is deleted if *B*_*i*,*j*_=2 holds before lines 7–9, i.e., there exists a path from *i* to *j* of length at least two. Since the graph is acyclic, this means that an indirect path from *i* to *j* without the direct edge (*ij*) exists. Thus, the edge (*ij*) is redundant and can be deleted. On the other hand, if an edge (*ij*) is redundant, an indirect path from *i* to *j* without the edge (*ij*) exists. This path must have length at least two. Consequently, we have *B*_*i*,*j*_=2 and the edge will be deleted.

For the correctness of the parallelised algorithm, we have to show that the steps performed in parallel are independent and do not interfere. For the parallel do-loop in lines 7–9 this is obvious as each iteration reads from and writes to its individual memory location. Analogously, all iterations of the inner loop of the Floyd-Warshall algorithm in lines 4–6 write to different memory locations. Furthermore, it cannot happen that for a fixed *k*_0_ the elements $B_{i_{0},k_{0}}$ and $B_{k_{0},j_{0}}$ read in the iteration for (*i*_0_,*j*_0_) were already changed by the iterations for (*i*_0_,*k*_0_) and (*k*_0_,*j*_0_), respectively, since both check $B_{k_{0},k_{0}}\geq 1$ which is never true since the graph is acyclic. The outer loop cannot be parallelised as it is crucial for the correctness of the Floyd-Warshall algorithm that all calculations of the *k*^th^ iteration are completed before the (*k* + 1)^th^iteration starts.

### Algorithm for weighted (cyclic) graphs

**Figure 5:**
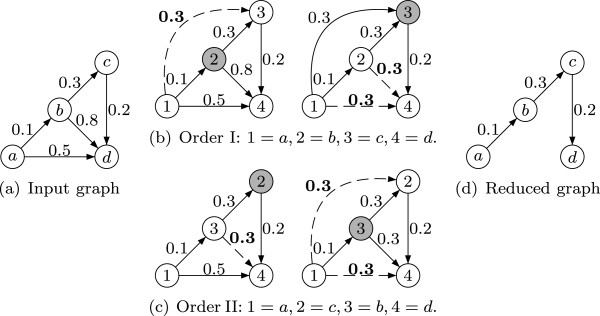
**An example how the Floyd-Warshall algorithm calculates the minimal transitive interaction uncertainties.** Consider order I of the nodes **(b)**: In the first iteration (*k*=1=*a*) nothing changes since no path via 1 exists as 1 has no incoming edge. In the next iteration (*k*=2=*b*) the path 1^→0*.*1^2^→0*.*3^3 with transitive interaction uncertainty, i.e., its maximal weight, 0*.*3 is found and this fact is memorised by adding 1^→0*.*3^3. Furthermore, the path 1^→0*.*1^2^→0*.*8^4 is considered, but as its transitive interaction uncertainty (0*.*8) is greater than the interaction uncertainty of the direct edge 1^→0*.*5^4, nothing changes. During the third iteration (*k*=3=*c*) the paths 2^→0*.*3^3^→0*.*2^4 and 1^→0*.*3^3^→0*.*2^4 (which corresponds to the path *a*^→0*.*1^*b*^→0*.*3^*c*^→0*.*2^*d*in the original graph) are found and since they have smaller (transitive) interaction uncertainties than the direct edges, these are changed to 2^→0*.*3^4 and 1^→0*.*3^4, respectively. In the last iteration (*k*=4=*d*), nothing changes since 4 has no outgoing edge and thus no path via 4 exists. Finally, all edges that were added or changed by the Floyd-Warshall algorithm, i.e., those for which an alternative path with a smaller transitive interaction uncertainty were found, are removed to obtain the weighted transitive reduction of the original graph as depicted in **(d)**. For order II of the nodes **(c)**, the algorithm works similar. Note that the correctness of the algorithm does not depend on the concrete order of the outer iteration (*k*) over the nodes, e.g., order I as well as order II produce finally the same result.

In order to obtain the TR of a weighted graph, according to the definition in Section “Background”, all edges (*i*,*j*)∈*E*for which an alternative path from *i* to *j* with a better transitive interaction uncertainty, i.e., *h*(*i*,*j*)<*w*(*i*,*j*), exists have to be removed. Consequently, the core of our algorithm is the calculation of the minimal transitive interaction uncertainties *h*(*i*,*j*) for every pair of nodes (*i*,*j*)∈*V*^2^. Again, we use a variant of the Floyd-Warshall algorithm whose pseudo-code description can be found in lines 3–7 of Algorithm 2. The matrix *B* is initialised with the original adjacency matrix (lines 1–2), i.e., *B*_*i*,*j*_=*w*(*i*,*j*), if (*i*,*j*)∈*E*, otherwise *B*_*i*,*j*_=⊤. Then, for increasing *k*=1,…,*n*all paths between each pair of nodes are successively considered that use only 1,…,*k* as intermediate nodes. The idea behind this is as follows: Let *B*_*i*,*j*_, *B*_*i*,*k*_, and *B*_*k*,*j*_contain the minimal transitive interaction uncertainty of all paths that go from *i* to *j*, from *i* to *k*, and from *k* to *j*, respectively, and that use only intermediate nodes up to *k*−1. Then, the minimal transitive interaction uncertainty of all paths from *i* to *j* that use intermediate nodes up to and including *k* is the maximum of the minimal transitive interaction uncertainties of the path segment from *i* to *k* and from *k* to *j* (line 5). If this value is smaller than the minimal transitive interaction uncertainty calculated so far, *B*_*i*,*j*_ is updated to it (lines 6–7). Figure 5 illustrates this principle. In the last step of the Floyd-Warshall algorithm, all paths with intermediate nodes up to *n*, which are in fact all paths, are considered. After this, the edges (*i*,*j*)∈*E* fulfilling the removal condition can be deleted by setting *B*_*i*,*j*_:=⊤ (lines 8–10) and the reduced matrix can be copied to and stored by the host (lines 11–12)

#### Algorithm 2.

**Pseudo-code description of parallelised transitive reduction of weighted, potentially cyclic, graphs**Requires: Adjacency matrix *A*, thresholds *t*_low_and *t*_up_

1: read *A* from input file

2: copy *A* from host to *B* on GPU

3: **for***k*=1,…, *n***do sequentially**

4: 

**for**$(i,j)\in \underset{–}{n}\times \underset{–}{n}$**do in parallel**

5: 

*viaK*:=max(|*B*_*i*,*k*_),|*B*_*k*,*j*_||

6: 

**if** (*B*_*i*,*j*_<0 or *B*_*i*,*j*_>*t*_low_) and *viaK*<|*B*_*i*,*j*_|**then**

7: 

*B*_*i*,*j*_:=−*viaK*

8: **for**$(i,j)\in \underset{–}{n}\times \underset{–}{n}$**do in parallel**

9: 

**if***isNegative*(*B*_*i*,*j*_) or *B*_*i*,*j*_≥*t*_up_**then**

10: 

*B*_*i*,*j*_: = T

11: copy *B* from GPU to *A* on host

12: write *A* to output file

However, two things are slightly more involved in the presented pseudo-code: First, to save memory, we use just one matrix *B* on the GPU for the whole computation. The problem is that if a path from *i* to *j* with a smaller transitive interaction uncertainty than the interaction uncertainty of the direct edge (*i*,*j*) is found, the original value in *B*_*i*,*j*_is overwritten. Thus, some necessary information to check the removal condition *h*(*i*,*j*)<*w*(*i*,*j*) is lost on the GPU. Yet, if this situation arises we already know that the edge (*i*,*j*) is to be deleted and we use the sign bit to indicate that. Consequently, instead of checking the original removal condition, we check for negative values in *B* and delete the corresponding edges in the post processing step (lines 8–10). Note that it is not possible to remove such edges (*i*,*j*) directly during the Floyd-Warshall algorithm since the absolute values for *B*_*i*,*j*_might be needed for later iterations. If, for example, in the second iteration (*k*=2=*c*) in Figure 5, case (c), the edge 3^→0*.*8^4 was directly removed and not replaced by 3^→0*.*3^4, the algorithm could not find in the next iteration (*k*=3=*b*) that 1^→0*.*1^3^→0*.*3^4 has a better (transitive) interaction uncertainty, namely 0*.*3, than the direct edge 1^→0*.*5^4.

The second aspect in the pseudo-code is the incorporation of thresholding. The upper threshold *t*_up_ is applied in line 9 (*B*_*i*,*j*_≥*t*_up_), ensuring that all edges with this or a bigger weight are always deleted. Due to the one-matrix representation, the lower threshold *t*_low_cannot be applied in this post processing fashion. Instead, every edge with this or a lower weight is skipped in line 6 (*B*_*i*,*j*_>*t*_low_) so that its original weight is preserved in *B*_*i*,*j*_.

#### Time complexity

The same as for Algorithm 1.

#### Correctness

We claim that the output of our algorithm is the thresholded weighted TR of the provided input, i.e., that in the end *B*_*i*,*j*_=*w*(*i*,*j*), for (*i*,*j*)∈*E*^*t*^, and otherwise *B*_*i*,*j*_=⊤ holds. Again, we prove the correctness of the sequential algorithm first, before we argue about the issues that come up with its parallelisation. Furthermore, we rely on the correctness of the Floyd-Warshall algorithm, i.e., that it correctly determines for each pair of nodes the minimum of the maximal weights of all paths between them.

First, we show that **all edges which are not in*E***^***t***^**are deleted from*B* (“*B*⊆*E***^***t***^**”)**. Initially, every matrix element *B*_*i*,*j*_ is non-negative. If a matrix element *B*_*i*,*j*_is changed for the first time by the Floyd-Warshall algorithm, then a negative value (−*viaK*) is written to it. This can happen only in two cases: i) For the edge (*i*,*j*)∈*E* that is not forced to be kept by the lower threshold, i.e., *w*(*i*,*j*)>*t*_low_, a path via some node *k* with a smaller transitive interaction uncertainty is found. Thus, this edge is to be deleted to obtain the TR. ii) A path from *i* to *j* via some node *k* was found and (*i*,*j*)∉*E*, i.e., no direct edge exists in the input graph. Of course, this edge must not be in the TR. Although the absolute value might change in later iterations if an even better path is found, once *B*_*i*,*j*_ becomes negative it stays negative. Consequently, in both cases, these edges will be removed in line 10. Note that for non-existing edges (*i*,*j*)∉*E* for which no path from *i* to *j* could be found the value of *B*_*i*,*j*_stays ⊤ during the whole algorithm. Finally, the post processing removes all of the remaining edges (*i*,*j*)∈*E* that are excluded from the TR by the upper threshold, i.e., *w*(*v*_*i*_,*v*_*j*_)≥*t*_up_, as for them the condition *B*_*i*,*j*_≥*t*_up_holds.

**Figure 6:**
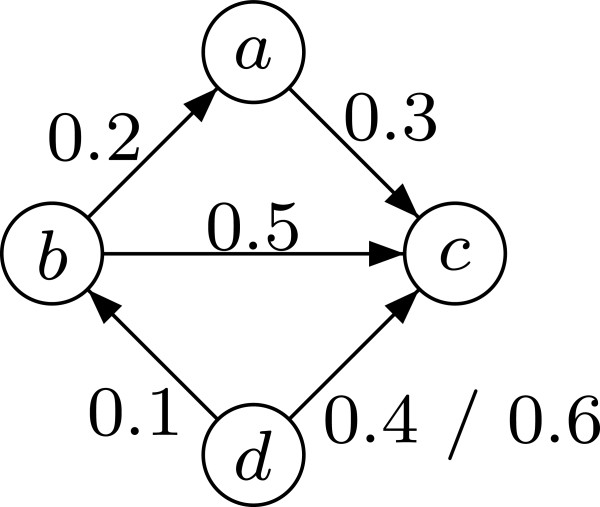
**Protecting edges by lower threshold *t***_**low **_**= 0.5 does not affect correctness.**

One might wonder whether the protection of edges by the lower threshold affects the correctness of the Floyd-Warshall algorithm. Consider, for example, the situation depicted in Figure 6 and assume *t*_low_=0*.*5: The edge *b*^→0*.*5^*c* is preserved although the better path *b*^→0*.*2^*a*^→0*.*3^*c* exists. Consequently, for the direct edge *d*→*c* the best alternative path *P*=*d*^→0*.*1^*b*^→0*.*2^*a*^→0*.*3^*c*with *W*(*P*)=0*.*3 is not found but only $P^{\prime }=d\overset{0.1}{\to }b\overset{0.5}{\to }c$ with *W*(*P*^*′*^)=0*.*5. Nevertheless, that still leads to a correct final result: If *w*(*d*,*c*)≤*t*_low_, e.g., 0*.*4, this edge is not removed even though the better alternative path *P* exists, since it is protected by the lower threshold. In contrast, if *w*(*d*,*c*)>*t*_low_, e.g., 0*.*6, this edge is not protected by the lower threshold and should be removed. This indeed happens since the path found (*P*^*′*^) is better.

It remains to show, that **the edges that are in*E***^***t***^**and their weights are preserved in*B* (“*B*⊇*E***^***t***^**”)**: As reasoned above, the matrix entry *B*_*i*,*j*_for an edge (*i*,*j*)∈*E* is changed only if a better path can be found *and* the edge is not protected by the lower threshold. Conversely, it stays unchanged and thus still holds the values *w*(*i*,*j*) for all edges (*i*,*j*)∈*E*for which no better path exists *or* that are protected by the lower threshold.

Finally, we have to argue that the **parallelisation of the algorithm** does not break its correctness. For the post processing in lines 8–10 this is definitely the case since every iteration reads only from and writes only to its individual memory location *B*_*i*,*j*_. As already discussed for the algorithm for unweighted acyclic graphs, it is crucial that the outer loop (line 6) of the Floyd-Warshall algorithm over *k* is executed sequentially. The inner loop (lines 7–8) however can be parallelised since for a fixed *k*_0_ of the outer loop every iteration (*i*_0_,*j*_0_) writes only to its own memory location $B_{i_{0},j_{0}}$ and reads only the values from $B_{i_{0},k_{0}}$ and $B_{k_{0},j_{0}}$ which are not changed throughout the whole *k*_0_th outer iteration. The values of $B_{i_{0},k_{0}}$ and $B_{k_{0},j_{0}}$ would be changed only if $\max\left(\left|B_{i_{0},k_{0}}\right|,\left|B_{k_{0},k_{0}}\right|\right)<\left|B_{i_{0},k_{0}}\right|$ or $\max\left(\left|B_{k_{0},k_{0}}\right|,\left|B_{k_{0},j_{0}}\right|\right)<\left|B_{i_{0},k_{0}}\right|$, respectively, holds. However, max(…,*r*,…)<*r*never holds for any r∈R.

## Results and discussion

We implemented our unweighted and weighted transitive reduction (TR) algorithms in the tools Cutter-u and Cutter-w, respectively. We performed two sets of experiments with these tools which were aimed at showing their scalability as well as their competitiveness and quality with regard to similar techniques.

### Scalability experiments with SynTReN generated graphs

In this set of experiments we used networks of various sizes (1,000, 2,500, and 10,000 nodes), with and without weights, as inputs for the TR algorithms. The unweighted networks were generated using the Directed Scale Free Graph algorithm [21] and the Erdős-Rényi algorithm [22]. To obtain the weighted graphs from them, the networks were simulated using the tool SynTReN[23]^a^. Using moderated *t*-test, the *p*-values for the interactions between each node pair were generated and used as edge weights.

The goal of this set of experiments was to test the scalability of Cutter-w and Cutter-u. We were interested in the speed-ups achieved by the parallel versions of our TR algorithms with regard to various sequential counterparts. All these experiments have been performed on (one core of) a personal computer with a 2.67 GHz Intel Core i7 CPU 920, and 12 GB RAM, running Ubuntu 10.10. For the parallel executions, we used Cuda 4.0 and Nvidia driver version 4.0 with an NvidiaGeForce GTX 480 graphics card with 1.5 GB VRAM and 480 streaming multi-processor cores, each running at 1.4 GHz.

For each graph size, we considered both acyclic unweighted and cyclic weighted graphs. We tested five implementations of TR algorithms. On acyclic unweighted graphs we applied Wagner’s algorithm [5], the sequential and the parallel implementations of Cutter-u. For the weighted cyclic graphs we compared the sequential and parallel implementations of Cutter-w.

The results of the algorithms are summarized in Tables 1 and 2. For the graphs of size 1,000 and 2,500 we considered five different graphs, whereas for the size 10,000 we used one scale-free graph^b^. For each graph the absolute runtimes were measured five times. The results for all runs on all graphs of the same size and type were very similar (with a spread less than 5%) and the averages are shown in Tables 1 and 2. In each table, the first three rows correspond to Wagner’s algorithm, the sequential Cutter-u, and the parallel Cutter-u, respectively. The last three rows give the relative speed-ups. One can see that the speed-ups increase with the graph size. For instance, for a weighted scale-free graph of 10,000 nodes, the parallel implementation is 92 times faster than the sequential one, which makes a difference between one and a half hours versus two minutes of reconstruction time.

**Table 1:** Scalability results for Wagner’s algorithm, sequential CUTTER, and parallel CUTTER on scale-free graphs

	**Unweighted**			**Weighted**		
**size**	**1,000**	**2,500**	**10,000**	**1,000**	**2,500**	**10,000**
**W [sec]**	2.14	34.33	2137.18	NA	NA	NA
**STR [sec]**	1.18	18.42	1186.39	7.52	120.21	10524.08
**PTR [sec]**	1.77	1.84	3.27	2.58	6.69	114.00
**STR vs. W**	1.82	1.86	1.80	NA	NA	NA
**PTR vs. W**	1.21	18.67	653.05	NA	NA	NA
**PTR vs. STR**	0.67	10.02	362.92	2.92	17.97	92.32

W = Wagner’s algorithm, STR = Sequential Cutter, PTR = Parallel Cutter, NA = not applicable (Wagner’s algorithm is restricted to unweighted graphs). The first block shows the absolute runtimes and the second gives the relative speed-ups.

**Table 2:** Scalability results for Wagner’s algorithm, sequential CUTTER, and parallel CUTTER on Erdős-Rényi graphs

	**Unweighted**		**Weighted**	
**size**	**1,000**	**2,500**	**1,000**	**2,500**
**W [sec]**	2.46	38.93	NA	NA
**STR [sec]**	1.20	18.55	5.86	91.12
**PTR [sec]**	1.74	1.82	2.44	6.37
**STR vs. W**	2.05	2.10	NA	NA
**PTR vs. W**	1.41	21.37	NA	NA
**PTR vs. STR**	0.69	10.18	2.40	14.31

W = Wagner’s algorithm, STR = Sequential Cutter, PTR = Parallel Cutter, NA = not applicable (Wagner’s algorithm is restricted to unweighted graphs). The first block shows the absolute runtimes and the second gives the relative speed-ups.

It is important to note that the structure and the density of an input graph has no significant effect on the performance of the TR algorithms. This can be concluded when comparing the results in Tables 1 and 2. Additional experiments that we performed with dense Erdős-Rényi graphs further confirm this^c^. In the case of dense Erdős-Rényi graphs the average runtimes for the weighted graphs of size 1000 and 2500 nodes are 2.50 and 7.36, which are comparable with their counterparts in Table 2. For the weighted graphs, this is not surprising, since each pair of nodes has a *p*-value assigned to it (e.g., recall from the Background section that “no edge” is represented by ⊤.). This means that for, e.g., a graph *G* with 10,000 nodes, the TR algorithms have to process 100,000,000 *p*-values, no matter what the edge density of *G* is. Thus, one can conclude that Cutter-w never requires significantly more than two minutes to reduce a graph of that size (Table 1).

### Quality experiments with the Dream 4 benchmark

In recent years, the Dialogue of Reverse Engineering Assessments and Methods (Dream) [10] challenge on in silico generated networks reconstruction has become an important benchmark. We tested Cutter-w, which implements our TR algorithm for weighted graphs, using the fourth challenge (Dream 4). The first goal of this set of experiments was to test the performance of Cutter-w compared to other state-of-the-art tools which had participated in the Dream 4 competition. Therefore, we compare Cutter-w with Cutter-u as well as with Transwesd[8], a tool which is also based on transitive reduction of weighted graphs. Since, besides the parallelisation, the main difference between Cutter-w and Transwesd is that the latter takes interaction signs into account, it was important to see how disregarding them affects the quality of the reduction in our approach. Our second goal was to investigate to which extent using interaction signs and the weights contributes to the reduction quality.

In our evaluation with the Dream suite we repeated the tests which were applied to Transwesd in [8]. In this way we wanted to ensure maximally fair comparison between the tools. Thus, we tested Cutter-w for the data set of the *InSilico_Size_100* subchallenge which can be downloaded from [24]. The data set consists of five networks of 100 nodes which are parts of real networks from E.coli and yeast. Noisy measurements representing steady state mRNA expression levels are generated in silico using GenNet Weaver[25]. We used only the data from the simulated knockout and knockdown experiments, ignoring the time series data. The original networks (gold standards) are known. (In the real competition they were provided, of course, later.) This makes the evaluation of the results much more objective than in the case of real networks, for which usually there is no consensus among the experts. To simulate the conditions of the real competition we tuned the parameters for Cutter-w (the thresholds) based on the results obtained with the Dream 3 challenge – in the same way like this was done for Transwesd in [8].

The network reconstruction was done in two steps, described in more detail in [8]. In the first step, the so-called *perturbation graph* is produced. In the second step, a (weighted) TR is applied on the graph to remove spurious edges. For all three reduction tools, Cutter-u, Transwesd, and Cutter-w we used as input the perturbation graphs produced by the first step of Transwesd. The edge weights were generated as conditional correlations from the knockout and knockdown data as in [8]. To generate those graphs we used the Matlab programs which were kindly provided by the authors of Transwesd. In the case of Cutter-u we simply disregarded both the weights and the signs in the perturbation graphs. Besides that, before applying Cutter-u, the possibly cyclic perturbation graph was transformed into an acyclic graph, whose nodes are strongly connected components, as described in Section “Background”.

The output files representing the reconstructed graphs were evaluated using the corresponding Matlab scripts provided by the Dream 4 challenge. The output files were formed by dividing the edges in three classes. The first class contains the accepted edges, the second, the edges accepted by the first step, but rejected in the second (TR) step, and the third one, the edges rejected already in the first step, i.e., already in the production of the perturbation graph. Each class is sorted based on the weights.

In the Transwesd tests in [8] the output files were composed by using only two classes of edges. The accepted edges after the second step were followed by the rejected ones. The edges within the same class were sorted in descending order according to their weights, like in our output files described above.

The results of our experiments are given in Table 3. For each of the five networks we give the results 1) without TR for the generated raw perturbation graphs, 2) with Cutter-u, 3) with TR using Transwesd and 4) with Cutter-w. The results with Transwesd are slightly different from the ones in [8]. This is due to a minor improvement in Transwesd as well as to the above mentioned different manner for producing the output files which were evaluated by the Dream 4 challenge. 

**Table 3:** Results with the networks of size 100 nodes from the DREAM4 benchmark

**Network + reconstruction method**	**TP**	**TN**	**FP**	**FN**	**AUROC**	**AUPR**
Network 1 (176 edges)						
-perturbation graph	114	9467	257	62	0.8851	0.5138
-perturbation graph + Cutter-u	107	9532	192	69	0.8848	0.5082
-perturbation graph + Transwesd	108	9547	177	68	0.8854	0.5366
-perturbation graph + Cutter-w	109	9582	142	67	0.8857	0.5475
Network 2 (249 edges)						
-perturbation graph	106	9389	262	143	0.7877	0.3577
-perturbation graph + Cutter-u	98	9411	240	151	0.7871	0.3455
-perturbation graph + Transwesd	92	9473	178	157	0.7874	0.3636
-perturbation graph + Cutter-w	85	9516	135	164	0.7874	0.3604
Network 3 (195 edges)						
-perturbation graph	93	9446	259	102	0.8490	0.3353
-perturbation graph + Cutter-u	91	9451	254	104	0.8488	0.3313
-perturbation graph + Transwesd	90	9543	162	105	0.8495	0.3574
-perturbation graph + Cutter-w	89	9563	142	106	0.8496	0.3673
Network 4 (211 edges)						
-perturbation graph	112	9403	286	99	0.8474	0.3932
-perturbation graph + Cutter-u	111	9418	271	100	0.8474	0.3938
-perturbation graph + Transwesd	101	9510	179	110	0.8478	0.4214
-perturbation graph + Cutter-w	96	9538	151	115	0.8477	0.4201
Network 5 (193 edges)						
-perturbation graph	66	9230	477	127	0.7667	0.1580
-perturbation graph + Cutter-u	66	9230	477	127	0.7667	0.1580
-perturbation graph + Transwesd	56	9409	298	137	0.7665	0.1653
-perturbation graph + Cutter-w	52	9495	212	141	0.7666	0.1661

TP = true positives, TN = true negatives, FP = false positives, FN = false negatives. AUROC = area under the receiver-operator characteristics curve and AUPR = area under the precision-recall curve are computed by the Dream 4 evaluation scripts.

AUPR (area under the precision-recall curve) and AUROC (area under the receiver-operator curve) are quite standard scoring metrics for binary classifiers, computed using the TP (true positives), TN (true negatives), FP (false positives), and FN (false negatives). For the definitions and a more detailed discussion on the scoring metrics for the Dream challenges see [10]. In all cases there is a clear gain from the weighted TR compared to the input perturbation graph. TR removes a significant number of false positives at the price of just a few false negatives. Cutter-w has on average 6% better AUPR than the perturbation graph. Also there is a clear improvement in the results with the weighted graphs compared to Cutter-u. Besides the significantly smaller number of false positives, Cutter-w has on average 7% better AUPR than Cutter-u with AUROCs being almost the same. The results of Cutter-w and Transwesd are quite similar. Cutter-w consistently generates significantly smaller number of false positives, which is compensated by Transwesd with more true positives, except for Network 1.

The overall Dream 4 score is calculated as $\mathrm{lo}g_{10}\sqrt{P_{1}\times P_{2}}$, where *P*_1_and *P*_2_ are the overall pAUPR and pAUROC values, respectively. The latter are obtained as geometric means of the individual pAUPR and pAUROC values of each of the five networks. Intuitively, the pAUPR and pAUROC are *p*-values that indicate how much our results, and in particular AUPR and AUROC, are better than randomly chosen networks. Some of these intermediate parameters were not included in the table, since they are summarized in the overall score. For the definitions of all of the above mentioned parameters see [10]. The overall Dream 4 score for Cutter-w is 70.93, for Transwesd it is 70.59 and for the unreduced perturbation graphs, however, it is only 68.27. With that we would have been ranked third behind the two best teams who had participated in the Dream 4 challenge at the time, with the scores 71.59 and 71.30, respectively. Considering that Cutter-w uses only part of the available data, i.e., only knockout and knockdown expression levels, this is quite an encouraging result. The improvement with regard to Transwesd is marginal. However, our intention was to show that disregarding the interaction signs, as we do in Cutter-w in practice does not result in loss of quality compared to Transwesd.

The results with Cutter-w can be even further improved by combining the methods of [8,14]. When generating the perturbation graph we use both knockout and knockdown data and the same parameters as for Transwesd. However, in the statistics we use instead of the wild type the average expression, like in [14]. For the transitive reduction step we generate the weights as before with Transwesd. As a result, we achieve the overall Dream 4 score 73.33, which is better than the above mentioned winning performance.

At first sight, the fact that by omitting the signs there is virtually no loss of quality of reconstruction can be paradoxical. However, in many cases the relative loss of information by the omission of the edge signs is compensated by the lower threshold *t*_low_ in the TR algorithm. We illustrate this with the following example. Consider a subnetwork of three nodes *a*, *b*, and *c*. Let there be a direct positive edge between *a* and *b* and an indirect negative influence path consisting of a negative edge between *a* and *c* and a positive edge between *c* and *b*. Being of opposite sign, the indirect influence via *c* does not explain (*a*,*b*). Nevertheless, an unsigned algorithm without a lower threshold will still remove edge (*a*,*b*). However, one can assume that the direct influence between *a* and *b* will result in a strong original weight (small uncertainty) on the edge (*a*,*b*). This will prevent an unsigned algorithm with a lower threshold to remove the edge.

## Conclusions

We presented parallel versions of algorithms for transitive reduction (TR) to reconstruct perturbation networks. The main improvement of our algorithms compared to the existing methods is the speed-up and scalability without loss of reconstruction quality. Moreover, our algorithms are applicable to both weighted and unweighted networks. The gain of the TR is significant since it mostly removes spurious direct interactions, which are overlooked by the first filtering step that produces the so-called perturbation graph.

We implemented our algorithms in the tool Cutter. Compared to similar approaches, like Transwesd, Cutter provides the same reconstruction quality, as measured by the Dream challenge benchmark. This is achieved despite the fact that we do not use signs of network interactions (note that this applies only to the TR phase; after reconstruction, the signs can be restored). The gain of this simplification is that our method can be efficiently parallelised, hence reconstruction is much faster and scalable. The algorithm in Transwesd is NP-complete, whereas our algorithms have a polynomial time complexity *O*(*n*^3^), where *n* is the number of nodes. This will be of utmost importance in the future when reconstruction methods will have to deal with whole genome knockouts or “hybrid” networks of genes, proteins and signaling molecules, i.e., with networks containing tens or even hundreds of thousands of nodes.

Since the TR algorithms depend on the threshold, fine tuning of this parameter might require several experiments. The advantage of obtaining the results within seconds or in the worst case minutes, instead of hours, can be very significant. Currently, it takes less than a couple of minutes to process with Cutter-w weighted graphs of 10,000 nodes and potentially 100 million edges.

Our weighted TR algorithm is independent of the nature of weights. Therefore, instead of correlations which were used for the Transwesd perturbation graphs, one can use *p*-values or other statistical estimates of the interaction strengths. Also, it might be interesting to explore alternative definitions of path weights. For example, the max operator, which was crucial in the definition of the path weight, can be replaced by any associative operator, like addition or multiplication.

Finally, although our approach is already quite effective despite its simplicity, it is worth considering combining it with other reconstruction methods.

## Availability and requirements

● **Project name:**Cutter

● **Project home page:**http://www.win.tue.nl/emcmc/cutter

● **Operating system(s):** Linux, Mac OS, Windows

● **Programming language:** C, CUDA

● **Other requirements:** CUDA

● **License:** none

● **Any restrictions to use by non-academics:** none

## Endnotes

^a^A special version of SynTReN, kindly provided by its authors, was used that allows simulation of single gene perturbation experiments. For more information see [26].^b^The reason for this is that, unfortunately, generating graphs of size 10,000 with SynTReN is very time and resource consuming.^c^These additional experimental results can be obtained at [26].

## Competing interests

The authors declare that they have no competing interests.

## Authors’ contributions

DB, WL, and PH conceived the project. DB came up with the idea to use GPUs for TR. All authors contributed to the theoretical part of the paper. DB, MO, and AW drafted the manuscript. MO and AW implemented the TR algorithms, performed the experiments and evaluated them. WL, AW, MO and DB implemented part of the support programs. All authors read and approved the final manuscript.
